# Chinese Consumer Assessment of Australian Sheep Meat Using a Traditional Hotpot Cooking Method

**DOI:** 10.3390/foods12051109

**Published:** 2023-03-05

**Authors:** Rachel A. O’Reilly, Liping Zhao, Graham E. Gardner, Hailing Luo, Qingxiang Meng, David W. Pethick, Liselotte Pannier

**Affiliations:** 1 Australian Cooperative Centre for Sheep Industry Innovation, Armidale, NSW 2351, Australia; 2 College of Science, Health, Engineering and Education, Murdoch University, Murdoch, WA 6150, Australia; 3 State Key Laboratory of Animal Nutrition, College of Animal Science and Technology, China Agricultural University, Beijing 100083, China

**Keywords:** lamb, yearling, eating quality, sensory, palatability, hotpot, meat standards Australia (MSA)

## Abstract

Hotpot is a widely popular cooking method for sheepmeat in China. This study measured the sensory responses of 720 untrained Chinese consumers to Australian sheepmeat cooked using a hotpot technique with methods based on Meat Standards Australia protocols. Shoulder and leg cuts of 108 lambs and 109 yearlings were scored on tenderness, juiciness, flavour and overall liking with linear mixed effects models used to analyse the influence of muscle type and animal factors on these scores. On average, shoulder cuts were more palatable than legs cuts for all sensory traits (*p* < 0.01) and lambs compared to yearlings (*p* < 0.05). Intramuscular fat and muscularity were identified as strong drivers of eating quality (*p* < 0.05), with greater palatability for both cuts as intramuscular fat increased (range 2.5 to 7.5%), and muscularity decreased (as measured through loin weight adjusted for hot carcase weight). Consumers were unable to detect differences between animal sire type and sex in sheepmeat hotpot. These findings suggest shoulder and leg cuts performed comparatively well in hotpot compared to previously tested sheepmeat cooking methods and emphasise the importance of balanced selection for quality and yield traits to ensure that consumer satisfaction is maintained.

## 1. Introduction

China is one of the world’s fastest growing economies, and with this growth, a corresponding increase in demand for sheepmeat products is expected, with wealthier consumers seeking variety in their protein sources [1,2]. While Chinese consumption of sheepmeat is much lower than beef and pork, domestic sheepmeat consumption far exceeds local production rates, providing a valuable market for Australian frozen sheep meat products [2]. Australian sheepmeat exports to China primarily include cuts such as breast, flap, manufacturing meat, neck and entire carcases, with a large proportion further processed into thin slice rolls used in hotpot restaurants [2]. Hotpot or huǒguō, as it is known in China, is one of the top three cooking methods for sheepmeat [3], and with hotpot restaurants accounting for approximately 20% of national restaurant revenue in China [2], it is important to understand the palatability of Australian sheepmeat when using this widely popular cooking method.

Hotpot traditionally involves cooking thinly sliced meat in a simmering soup of various base stocks, spices and vegetables [3,4]. These thinly sliced rolls of meat are often sold frozen and fabricated from either whole-tissue or a restructured “block” consisting of layers of lean tissue and fat [3], generally sourced from boneless shoulder and leg sheepmeat cuts. Chinese consumers have shown a preference for a higher degree of visible fat content in their sliced hotpot beef [4], and it is well reported that fat improves flavour profiles, with lipids providing species specific flavours that increase with greater volumes present [5]. In addition, lipids create a lubricative effect, thereby increasing the perceived juiciness of a product [6,7]. Considering the positive association between fat and eating quality, shoulder cuts may prove more palatable for hotpot, with higher fat to lean ratios reported in the lamb forequarter compared to the hindquarter [8].

Palatability can be described through the sensory attributes of tenderness, juiciness, liking of flavour and overall liking [9], with a positive eating experience improving consumer satisfaction and subsequent repurchase intent [10]. The Meat Standards Australia (MSA) system is underpinned by these eating quality attributes and has been used to evaluate large numbers of untrained consumers both domestically and internationally using a variety of cuts and cooking methods [11,12,13,14,15,16]. In beef, Japanese and Korean consumer sensory scores are influenced by cooking method, muscle type and slaughter conditions when using traditional cooking methods of yakiniku, shabu shabu and barbeque for local and Australian products [12,13]. Similarly, previous research in sheepmeat [17] demonstrated that untrained Chinese consumers could also differentiate animal factors during eating quality tests, preferring lamb rather than yearling, loin cuts rather than topside, and samples from Merino and Maternal sired lambs rather than Terminal. In addition, increasing intramuscular fat (IMF) levels had a positive influence on sensory scores for sheepmeat [18]. However, all previous findings in Chinese consumers were from a grill cooking method rather than traditional hotpot. In addition, a positive association between eating quality and phenotypic factors of higher IMF levels, greater fatness and lower muscling has also been reported in Australian consumers [14,19,20]. Whether thinly sliced shoulder and leg cuts are as heavily influenced by these carcase attributes is unknown.

This study developed an MSA sheepmeat hotpot testing protocol to examine the sensory responses of Chinese consumers to Australian lamb (no erupted permanent incisors) and yearling (2–4 erupted permanent incisors) shoulder and leg cuts cooked using a traditional Chinese hotpot cooking technique. We hypothesised that shoulder cuts would score greater than leg cuts, particularly for flavour and juiciness, and that a preference for Merino and Maternal sired animals and lambs would be evident rather than Terminal. In addition, we hypothesised that scores would improve in carcases with decreasing loin weight and increasing carcase fatness.

## 2. Materials and Methods

### 2.1. Carcase Details and Muscle Collection

Lambs and yearlings (*n* = 217) utilised in this study were sourced from the Meat and Livestock Australia genetic resource flocks located in Katanning, WA, and Kirby, NSW. Details of this flock design have been published elsewhere [21,22]. Lambs and yearlings from the Katanning site were on average, 341 days and 701 days old at slaughter. Lambs and yearlings sourced from Kirby were on average, 351 days and 716 days old at slaughter. Consistent for both sites, lambs (females and wethers) were sired by Maternal (Border Leicester and Dohne Merino), Merino (Merino and Poll Merino) and Terminal (Poll Dorset, Suffolk, Texel and White Suffolk) sires, while yearlings were strictly wethers and progeny of Merino (Merino and Poll Merino) sires. Animals were slaughtered at commercial abattoirs licenced for export to China and processed according to resource flock protocols [21], including application of medium-level electrical voltage, trimming according to AUSMEAT specifications [23], and chilling for 24 h at 3–4 °C to allow optimal pH decline [24]. Entire boneless shoulder (AUS-MEAT 5050) and leg (AUS-MEAT 5070) cuts were collected from each carcase at 24 h post-slaughter [23], vacuum packaged and aged for 10 days at 2–3 °C prior to frozen (−20 °C) export to China. All cuts were handled under commercial processing conditions adhering to Australian food safety standards for exports. A total of 216 shoulder and 216 leg cuts were collected from 217 carcases, of which two carcases did not have both cuts available (*n* = 2).

### 2.2. Carcase Compositional Measurements

The hot carcase weight (HCWT) of each animal was recorded immediately after slaughter. Each carcase was split at the 10th/11th thoracic rib 24 h post-mortem to collect the full *longissimus lumborum* (LL) from the saddle region of each carcase (AUS-MEAT 4880) [23]. The subcutaneous loin fat was trimmed and weighed (LLFAT), and the remaining lean muscle tissue was weighed (LLWT). IMF, expressed as a percentage, was measured by sampling 40 g of lean LL tissue. Each sample was freeze dried using a Cuddon FD 1015 freeze dryer (Cuddon Freeze Dry, Blenheim, New Zealand) and IMF was measured using near infrared spectroscopy (NIR) in a Technicon 450 Infralyser Spectrophotometer (19 wavelengths). NIR readings were validated against Soxhlet extraction for determination of chemical fat content [25]. Detailed measurement protocols for IMF have previously been reported by Perry et al. [26].

### 2.3. Sample Preparation and Sensory Testing

This research was approved by the Murdoch University Human Research Ethics Committee (Project No. 2016/015).

Tasting sessions were conducted in Beijing, China, at the China Agricultural University in 2016. As such, participants were localised to the community surrounding the university. A total of 720 consumers were recruited through university social networks to participate in one of twelve hotpot sessions, with recruitment requesting participants to be aged between 18–70 years old and consuming sheepmeat at least once per fortnight. Prior to starting the tasting session, demographic data was collected for each participant, including questions on age, gender, income and occupation. The survey design is described in detail by O’Reilly et al. [27]. Each consumer tasted and scored six samples (3 shoulder and 3 leg) for tenderness, juiciness, liking of flavour and overall liking on 100 mm scale lines, consistent with standard MSA testing protocols [11,28]. Scale lines were anchored with “not” (allocated as a 0 score) and “very” (allocated as a 100 score) preceding the eating quality trait for tenderness and juiciness and “dislike extremely” and “like extremely” for liking of flavour and overall liking. While the MSA tasting session structure was consistent with previous grilled sheepmeat sensory research within China and Australia [11,14,17], a new MSA traditional Chinese hotpot cooking method was developed for this study.

Within 24 h of each sensory session commencing, shoulder and leg cuts were trimmed to a uniform 50 × 50 × 100 mm block while partially frozen, and ten 1.6 mm slices were prepared from each block using a commercial meat slicer. The 10 slices per cut were uniformly spread across the entire block to represent slices across the entire cut and stored in the fridge at 0–5 °C in designated cooking order until the start of each session.

Within one hotpot session, a total of 36 cuts were tested, with an appropriate cooking and consumption order allocated by a 6 × 6 Latin square design [29]. Each slice was cooked in unseasoned boiling water in self-contained bain-marie pots (one pot for the 10 slices of the same cut) for 2 min before prompt service to the consumer. The 10 slices per cut were served to 10 different consumers to obtain 10 sensory scores per cut. A total of 4320 sensory responses were recorded across the 12 tasting sessions conducted over a one month period.

### 2.4. Statistical Analysis

Sensory scores of tenderness, juiciness, liking of flavour, and overall liking were analysed using linear mixed effects models in SAS (SAS Version 9.1, SAS Institute, Cary, NC, USA). Consumer variation was accounted for with the inclusion of all 10 consumer responses for each cut. To test animal factors and muscle type, the base model for each sensory trait included fixed effects of muscle type (shoulder, leg), sire type-age class as one single term (Maternal lamb, Merino lamb, Terminal lamb and Merino yearling), sex within sire type-age class (female and wether lambs with each sire type group, and Merino wether yearling), and site (Katanning, Kirby). Relevant first-order interactions between fixed effects were tested and non-significant terms removed using stepwise regression. The Satterthwaite function was included in all models to approximate degrees of freedom. Random terms included animal identification within sire and consumer identification within a hotpot session. Partial correlation coefficients were also calculated between the four sensory traits within each cut and between the shoulder and leg cuts using multivariate analysis of variance in SAS (SAS Version 9.1, SAS Institute, Cary, NC, USA). Significant class variables of the base models were included in the independent part of the multivariate model.

In order to test whether differences in fixed effects could be explained by carcase composition differences, phenotypic covariates of IMF, LLFAT, LLWT and HCWT were included in the base models mentioned above to evaluate their association with tenderness, juiciness, liking of flavour and overall liking. To test the association between muscularity or carcase fatness with eating quality, LLWT or LLFAT were used as indicators. In each instance, hot carcase weight was included in the models in order for these terms to act as proxies for carcase muscling and fatness rather than just acting as simple correlates of carcase weight. As IMF was only measured in the loin muscle, the impact of IMF on eating quality traits for shoulder and leg cuts was assessed using the loin IMF.

## 3. Results

### 3.1. Data Structure and Descriptive Statistics

The number of lambs and yearlings included in the base model analyses for each category is detailed in Table 1. Of the 217 carcases included in the analyses, 215 were represented by both shoulder and leg cuts. Age class comparisons were made across Merino groups given that yearlings were entirely Merino sired. Given low Maternal and Merino lamb numbers within each site, site comparisons were only made across Terminal lamb and Merino yearling groups. Sire type and sex comparisons were made only within the lamb groups, with the acknowledgement of low animal numbers within the Maternal and Merino sired classes. Table 2 presents the unadjusted mean and range for the phenotypic covariates of HCWT, LLFAT, LLWT and IMF percentage, which were included as covariates in the base models.

Of the 720 Chinese participants recruited, 61% were female, with the majority aged between 18–25 years old (52%), followed by those over 40 years of age (25%). The participants comprised largely of the lowest income bracket of ≤ CNY 24,000 per annum (49%), with equal numbers in CNY 24,000 to CNY 36,000 and CNY 36,001 to CNY 60,000 income categories (13%). Consumers identified themselves primarily as students (30%), professionals (19%), and labourers (13%).

### 3.2. Model Outcomes

The outcomes of the base models for tenderness, juiciness, liking of flavour and overall liking are presented in Table 3. The variance accounted for by these models was 33%, 33%, 35% and 35% for tenderness, juiciness, liking of flavour and overall liking, respectively. The predicted mean eating quality scores for the significant effects of sire type-age class and sire type-age class by site between shoulder and leg cuts are detailed in Table 4. Results are largely indicated as the magnitude of difference in sensory score units of 0–100, and where the effect of covariates are included in base models, results are presented across the range for each covariate trait, as determined by the mean ± two standard deviations for each trait.

#### 3.2.1. Impact of Cut, Age Class, Sire Type and Site on Sensory Scores

The shoulder cut had greater palatability compared to the leg cut for all sensory traits, with total average shoulder scores being 64.0, 62.7, 66.7 and 67.8, compared to the leg scores at 60.5, 60.0, 63.6 and 64.1 for tenderness, juiciness, liking of flavour and overall liking, respectively (*p* < 0.01; Table 3 and Table 4). For tenderness, the magnitude of this cut effect differed between sire types, with the greatest difference in cuts detected in Maternal lambs (5.7 ± 2.6; *p* < 0.05), followed by Terminal lambs (3.8 ± 0.9; *p* < 0.001), with no significant cut differences observed within wether Merino lamb or yearling subgroups.

Age class had a significant effect on palatability, with Merino yearlings on average scoring lower for all sensory traits compared to Merino lambs (*p* < 0.05; Table 3). Comparisons could only be made between Merino groups, as yearlings were entirely Merino sired. On average, Merino lambs had higher eating quality than yearlings, with increases of 6.6, 4.2, 3.9 and 4.5 units for tenderness, juiciness, liking of flavour and overall liking, respectively (Table 4). For tenderness, the age class difference varied between cuts, with lamb shoulders scoring 8.0 units greater than yearling (*p* < 0.001; Table 4), whereas lamb legs scored 5.1 units greater compared to yearling legs (*p* < 0.05; Table 4). In addition, the age class effect varied by site with Katanning Merino lambs scored greater than Merino yearlings by 4.4 flavour and 5.3 overall liking units, compared to Kirby sourced animals, whereby no age effect was evident.

Comparisons across all the sire type-age class groups demonstrated that Terminal lambs on average were also preferred to Merino yearlings for tenderness, juiciness, flavour and overall liking by up to 3.8 scores and Maternal lambs to Merino yearlings for tenderness by 5.6 scores (*p* < 0.05; Table 3 and Table 4).

Overall, site had a significant effect on palatability, with Katanning sourced animals scoring higher than those obtained from Kirby for tenderness, juiciness and liking of flavour with average increases of 1.7, 2.4 and 2.5 units, respectively (*p* < 0.05; Table 3). Comparison across sites demonstrated that Terminal lambs at Katanning had higher liking of flavour and overall liking scores compared to animals from Kirby, with increases of 5.8 and 5.2 sensory units (*p* < 0.001; Table 4). There was no difference between the sensory scores of Merino yearlings between the two sites.

Sire type comparisons could only be made within the lamb group in this dataset, given yearlings were strictly Merino sired. There were no significant differences observed between Maternal, Merino and Terminal sired lamb sensory scores when testing shoulder and leg cuts using a hotpot cooking technique. Similar to sire type, true sex comparisons could only be made within lambs, and there was no significant effect of animal sex on any sensory traits within this study.

#### 3.2.2. Impact of Intramuscular Fat on Sensory Scores

**Figure 1:**
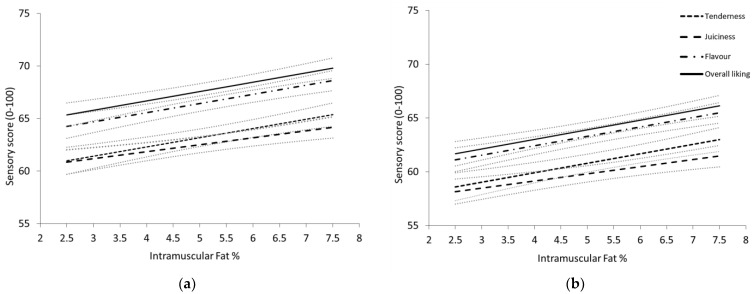
Relationship between intramuscular fat (%) of the loin muscle and tenderness, juiciness, liking of flavour and overall liking scores of the (**a**) shoulder cut; (**b**) leg cut. Lines represent the least square means of each cut (±SE) across an increasing intramuscular fat % range.

Loin IMF percentage had a significant positive relationship with tenderness, juiciness, liking of flavour and overall liking consumer scores for both the shoulder and leg cuts (*p* < 0.05). Across the loin IMF range of 2.5% to 7.5%, sensory scores increased by 4.4, units for tenderness, liking of flavour and overall liking and 3.3 units for juiciness (Figure 1). When IMF percentage was included in the base models (Table 2), site and cut by sire type-age class no longer had a significant effect on tenderness scores, and site by sire type-age class was non-significant for flavour and overall liking results.

#### 3.2.3. Impact of Carcase Compositional Indicators on Sensory Scores

**Figure 2:**
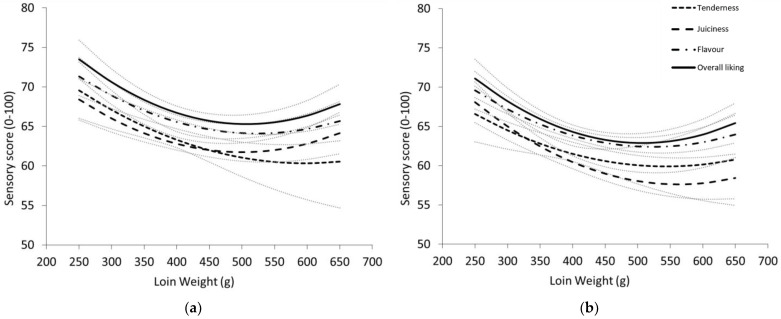
Relationship between loin weight (g) adjusted for hot carcase weight for tenderness, juiciness, liking of flavour and overall liking scores of the (**a**) shoulder cut; (**b**) leg cut. Lines represent the least square means of each cut (±SE) across an increasing loin weight range.

Muscularity measured through LLWT adjusted for HCWT had a significant and generally negative effect on all sensory traits (*p* < 0.05; Figure 2). Increasing LLWT reduced average sensory scores for all traits up to 500 g, beyond which there was little change. Flavour liking and overall liking decreased by 7.2 and 8.2 units, while tenderness and juiciness varied across cuts, with shoulder scores reduced by 9.1 and 6.7 units, and leg cuts by 6.5 and 10.0 units. For tenderness only, the impact of LLWT varied between sire type-age class groups and cuts. Following the general trend, tenderness decreased for Terminal lamb cuts, Merino lamb shoulders and yearling legs from 5.8 to 10.4 units. In contrast, Merino lamb legs and yearling shoulders increased in tenderness by 5.3 and 9.2 units. When LLWT was included in the base models (Table 2), there was no longer a significant difference between the average sire type-age class groups for flavour and overall liking.

Whole carcase adiposity, represented by LLFAT adjusted for HCWT, had a significant effect only within juiciness scores at the Katanning site (*p* < 0.01). Increasing LLFAT from 50 to 650 g improved the juiciness of Katanning sourced animals by 12.1 units. Inclusion of LLFAT within the juiciness base model (Table 2) removed the significant effect of sire type-age class.

### 3.3. Correlations between Sensory Traits and Cuts

The partial correlation coefficients between tenderness, juiciness, liking of flavour and overall liking within the shoulder and the leg cuts are presented in Table 5. All correlations were high (*p* < 0.01), with the strongest association between liking of flavour and overall liking. Between muscle partial correlation coefficients were not significant.

## 4. Discussion

### 4.1. Impact of Cut on Sensory Scores and Correlations

In agreement with our hypothesis, shoulder cuts consistently scored higher than leg cuts using a hotpot cooking method; however, the greatest improvements were found for overall liking (3.7 unit increase) and tenderness (3.5 unit increase), followed by liking of flavour and juiciness (2.7 unit increase) (*p* < 0.01; Table 3 and Table 4). These findings agree with eating quality observations in grilled and roasted lamb muscles, whereby Australian consumers in general scored forequarter muscles more favourably than hindquarter muscles [11,15,16]. Across all sensory traits, roasted forequarter sensory scores ranged from 56.2 to 65.3, compared to easy carve legs, ranging from 46.9 to 60.8 [15,16]. Similarly, grilled *Serratus ventralis* scores ranged from 70.1 to 73.4 compared to rumps and topsides, ranging from 37.3 to 67.1 [11]. In comparison to the Australian studies [11,15], hotpot shoulder and leg sensory scores were slightly greater than when using a roast cooking method, but shoulders were scored lower than if they were grilled (shoulder scores 62.7 to 67.8; leg scores 60 to 64.1). This indicates that hotpot is an equivalent, if not more favourable, cooking method compared to roast for these cuts, and shows potential for inclusion in the sheepmeat MSA cut by the cooking method eating quality grid [19]. However, it should be considered that a traditional cooking method was used for the culturally relevant group, so whether the same results would be evident using hotpot with Australian consumers is unknown. Nevertheless, the ability of Chinese consumers to discern eating quality differences between cuts in this study aligns with previous testing of grilled loin and topside muscles, where Chinese consumers routinely scored the loin higher than the topside [17]. Notably, cut accounted for less variation in the hotpot models compared to a roast cooking method using the same cut type [16] and corresponded with a smaller magnitude of difference between the shoulder and leg eating quality scores. The range in hotpot scores was 2.7 to 3.7 units compared to 4.2 to 10.3 units for the roast cooking method [16]. It is plausible that the intrinsic factors of various muscle types that respond to cooking are removed through physical disruption of the muscle structure with the thin slicing employed for hotpot cooking. For example, tenderness is reduced through collagen fibre shrinkage during cooking in muscles of higher connective tissue content [30]; thus, differences expected based on muscle composition would be less pronounced using this cooking method. These results reinforce the value of using MSA cut-by-cook testing protocols in the assessment of palatability of sheepmeat products for international consumer groups, with numerous similar comparisons in beef for a variety of cuts and cooking methods [13,31,32,33].

Shoulder and leg cuts both demonstrated strong correlations between sensory scores of tenderness, juiciness, liking of flavour and overall liking (Table 5). The strongest correlations were observed between liking of flavour and overall liking for both cuts at 0.92, which aligns with previous findings in China, with r values of 0.86 and 0.90 reported for grilled loin and topside [17]. This relationship is consistent with international research findings in lamb for Australian and American consumers [14,34] and in beef for French, Irish, Japanese and Polish consumers [31,35]. This demonstrates that, regardless of cultural background, consumer eating quality scores are strongly associated with one another and should be interpreted with a degree of caution due to the consumers’ inability to differentiate between the traits [36,37]. In agreement with O’Reilly et al. [17] and Thompson et al. [11], correlations between the different cuts were low and non-significant for all sensory traits, thus emphasising the importance of testing the palatability of each individual cut when developing new cooking methods.

### 4.2. Impact of Age Class, Sire Type and Site on Sensory Scores

In support of our hypothesis, Merino lambs scored higher than Merino yearlings for tenderness, juiciness, flavour and overall liking. In addition, there was a greater age class improvement observed for tenderness in the shoulder compared to the leg. Comparisons to previous research are challenging due to the novel cooking method and the use of different cuts in this study. However, the results generally align with previous research demonstrating improved palatability in younger animals for some sensory traits and variation across cuts [14,17,38,39]. For untrained Australian consumers tasting lamb and yearling samples, increases of 5 to 10 units were observed in grilled lamb topsides for tenderness, liking of flavour and overall liking (1337 consumers; 185 lambs & 206 yearlings) [40]. Furthermore, roasted lamb topsides and *M. biceps femoris* (outsides) extracted from the easy carve leg had improved sensory scores compared to yearlings, ranging from 4.5 to 7.9 and 10.2 to 16.1 units for tenderness, juiciness and overall liking (480 consumers; 18 lambs & 18 yearlings) [38]. Conversely, for both studies, minimal or no significant improvements were reported in the eating quality of loins in lambs compared to yearlings across all traits [38,39]. Perhaps more comparable would be the results of untrained Chinese consumer testing of grilled sheepmeat, with Merino lamb loin and topsides scoring 8.4, and 6.9 tenderness units, and 4.7, and 5.1 overall liking units greater than Merino yearlings (720 consumers; 112 lambs & 106 yearlings) [17]. This is similar to tenderness scores in the current study, with Merino lamb shoulders and legs scoring 8.0 and 5.1 units greater than yearlings (*p* < 0.001). While discrepancies exist depending on cut, cooking method and cultural background, it is clear that age at slaughter does have some effect on eating quality between lambs and yearlings. Declining eating quality associated with animal age is attributed to several factors, including the build-up of undesirable flavours in fat deposits [41] and increased cross-linking between collagen fibres resulting in reduced tenderness as the animal ages [40].

Contrary to our hypothesis, sire type did not have an impact on the average palatability of shoulder and leg cuts using a hotpot cooking method. These results are in contrast to previous findings for Australian and Chinese consumers [14,17], with Merino sired animals reported to have higher eating quality than their Maternal and Terminal sired counterparts for Australian consumers, with increases in all sensory traits ranging from 2.9 to 5.2 units [14], while Chinese consumers reported tenderness improvements specific to Merino topsides (9.3 units) [17]. Given the latter studies have utilised grilled sheepmeat and sire type influenced only tenderness in topsides for Chinese consumers, it is perhaps unsurprising that sire type differences were not detected in the thinly sliced hotpot samples. In addition, Maternal and Merino lamb numbers were low in this study (11 and 16), suggesting these results should be interpreted with caution, and further testing with increased lamb numbers may be warranted.

Katanning sourced carcases were preferred to those obtained from Kirby for sensory traits of tenderness, juiciness and liking of flavour. This site effect combines flock composition (different sire type proportions), nutritional management and slaughter conditions. Site, flock and kill group differences are commonly reported as a source of variation in eating quality studies and can encompass a wide range of extrinsic factors leading up to slaughter and processing, including but not limited to lifetime nutrition, inclement weather, stock handling, and chilling regimes [14,17,39]. Cross comparisons of the sites within this study identified Katanning Terminal sired lambs had greater flavour and overall liking scores than Kirby animals, up to a difference of 5.8 units, which is of a reasonable magnitude and likely a driver of improved site results given that Terminal lambs comprised approximately forty percent of the Katanning cohort (Table 2). Levels of IMF for this Terminal lamb group provide a plausible explanation for palatability improvements, particularly given the effect was no longer significant when IMF was included in base models.

### 4.3. Impact of Intramuscular Fat on Eating Quality

Confirming our hypothesis, increasing intramuscular fat had a significant positive effect on all sensory traits for shoulder and leg cuts using the hotpot cooking method. For every one percent increase in IMF, consumer scores increased by 0.9 units for tenderness, liking of flavour and overall liking and 0.7 units for juiciness. These results are lower than previously demonstrated consumer responses to IMF in grilled sheepmeat, with Chinese scores increasing at a rate of 1.8 units for all traits except for juiciness [18] and Australian scores increasing from 1.2 to 2.4 units across all sensory traits [14,18,20]. While the magnitude of effect was lower than previous findings, the positive IMF influence aligns well with prior studies demonstrating its strong association with eating quality in sheepmeat [14,20]. The thin slicing of the hotpot meat did not entirely negate the effect of IMF on palatability, indicating selection for IMF is still important for carcases destined for the hotpot market. The inclusion of IMF in base models rendered several factors non-significant. Average site differences were eliminated for tenderness, along with the flavour and overall liking preference for Katanning sourced Terminal lambs compared to Kirby. Katanning Terminal lambs had a much higher IMF range (3.8 to 10.6%) than Kirby (3.3 to 6.3%); thus, it is reasonable to suggest IMF as a primary driver of these differences. In addition, IMF explained the greater tenderness discrepancies between lamb and yearling shoulder cuts compared to legs.

### 4.4. Impact of Muscularity and Whole Carcase Adiposity on Sensory Traits

Validating our hypothesis, muscularity (LLWT adjusted for HCWT) appeared to have a negative influence on all sensory traits. Across an increasing 400 g range, eating quality declined 6.5 to 10.0 scores (*p* < 0.05; Figure 2). These results were comparable to Pannier et al. [14], who observed eating quality reductions from 3.4 to 7.0 units for loin samples across an increasing 360g loin weight range, and 4.5 to 9.3 unit declines in palatability of the topside for a 480 g topside weight range. The impact varied by cut for tenderness and juiciness, with a surprising effect observed in Merino yearling shoulders and Merino lamb legs with increasing scores across the range, instead of the expected decline. This is somewhat difficult to explain, as each cut was fabricated to the same dimensions for slicing. However, it is conceivable that the greater lean-to-fat ratio observed in higher muscling animals could have prompted perceptions of better value with “larger” servings, creating greater satisfaction for Chinese consumers. Inclusion of IMF in these models did not negate the LLWT effect in any covariate models.

Contrary to expectations, whole carcase adiposity as measured by LLFAT adjusted for HCWT did not have a significant impact across all eating quality traits or shoulder cuts specifically. This is despite the supposition that shoulder cuts would be preferred to legs based on greater intermuscular fat levels [8] and the positive association between fat and sensory attributes [6,7]. Furthermore, LLFAT only had a significant effect for juiciness within the Katanning site. Correction for the IMF trait yielded LLFAT non-significant, suggesting results were driven through greater IMF levels in Katanning animals rather than intermuscular fat.

## 5. Conclusions

While Chinese consumers demonstrated an eating quality preference for thinly sliced shoulder muscles using a traditional hotpot cooking method, both shoulder and leg cuts scored reasonably well compared to equivalent muscles cooked using MSA grill and roast cooking methods, proving hotpot to be a highly acceptable cooking technique for these products. IMF was a strong driver of eating quality for all traits, and while the effect was lower in magnitude than previously reported for Chinese and Australian consumers testing grilled sheepmeat, it explained many of the site and sire type differences observed for tenderness, flavour and overall liking. Furthermore, increasing carcase muscularity saw a steady decline in all sensory scores, highlighting that quality and yield attributes can be detected by Chinese consumers, even at the level of a 1.6 mm slice. Thus, producers should be mindful of heavy selection for lean meat yield and strive for higher IMF levels given that balanced selection will reap positive returns even for products destined for further manufacturing channels, in this case thinly sliced hotpot rolls.

## Figures and Tables

**Table 1:** Number of lambs and yearlings included in the base models (n = 217), categorised by site, age class, sire type and sex.

Site	Age Class	Sire Type	Sex	Count
Kirby	Lamb	Maternal	F	3
			M	3
		Terminal	F	22
			M	19
		Merino	F	1
			M	2
	Yearling	Merino	M	70
Katanning	Lamb	Maternal	F	2
			M	3
		Terminal	F	27
			M	13
		Merino	F	10
			M	3
	Yearling	Merino	M	39

**Table 2:** Number of sires, number of animals (*n* = 217) and their unadjusted mean ± SD (min—max) for the phenotypic traits included in base model analyses.

Age Class	Sire Type	No. of Sires	No. of Lambs	Hot Carcase Weight (kg)	Loin Fat Weight (g)	Loin Weight (g)	IMF %
				Mean ± SD (min—max)	Mean ± SD (min—max)	Mean ± SD (min—max)	Mean ± SD (min—max)
Lamb	Maternal	8	11	25.2 ± 3 (20.5–29.7)	355.8 ± 147.4 (118–550)	429.5 ± 75.1 (296–531)	5.9 ± 1.1 (4.4–8)
	Merino	10	16	22.1 ± 3.7 (16.6–30.4)	215.4 ± 100.5 (111–435)	369.8 ± 65.4 (263–483)	5.3 ± 1.2 (3.3–7.7)
	Terminal	42	81	27.1 ± 5.6 (17.4–39.5)	410.2 ± 211.2 (129–939)	498.5 ± 129.9 (259–784)	5.3 ± 1.1 (3.3–10.6)
Yearling	Merino	47	109	21.4 ± 3 (15–29.9)	149.9 ± 68.9 (44–386)	374.2 ± 62.1 (243–544)	5.1 ± 1.3 (2.4–8.9)

**Table 3:** *F*-values, NDF and DDF for base linear mixed effects models for predicted eating quality scores of tenderness, juiciness, liking of flavour and overall liking.

Effect	Tenderness		Juiciness		Flavour		Overall liking	
	NDF, DDF	*F*-value	NDF, DDF	*F*-value	NDF, DDF	*F*-value	NDF, DDF	*F*-value
Cut	1, 3654	15.8 **	1, 3419	24.61 **	1, 3424	37.46 **	1, 3420	51.79 **
Sire type-age class	3, 197	9.76 **	3, 190	4.23 *	3, 194	4.31 *	3, 196	5.8 **
Site	1, 193	4 *	1, 187	10.6 *	1, 201	4.48 *	1, 203	2.53
Cut × Sire type-age class	3, 3729	2.7 *	-	-	-	-	-	-
Site × Sire type-age class	-	-	-	-	3, 202	3.16 *	3, 204	3.21 *

NDF, DDF: numerator and denominator degrees of freedom;-: not present in final models after stepwise regression; *: *p* < 0.05: **: *p* < 0.001.

**Table 4:** Least square means ± SE for effects of sire type-age class and sire type-age class by site between shoulder and leg cuts for degree of tenderness, juiciness, liking of flavour and overall liking.

Site	Age Class	Sire Type	Cut	Tenderness	Juiciness	Flavour	Overall Liking
Kirby	Lamb	Maternal	shoulder	68.5 ± 3.1	64.3 ± 2.8	68.3 ± 2.7	69.7 ± 2.7
			leg	61.4 ± 3.1	56.9 ± 2.8	62.4 ± 2.7	63.2 ± 2.7
		Merino	shoulder	73.6 ± 5.3	69.8 ± 4.8	70.9 ± 4.5	72.2 ± 4.6
			leg	66.3 ± 4.4	61.3 ± 4	61.9 ± 3.8	62.8 ± 3.8
		Terminal	shoulder	63.0 ± 1.2	62.1 ± 1.1	65.2 ± 1.1	66.5 ± 1.1
			leg	56.8 ± 1.2	56.7 ± 1.1	59.5 ± 1.1	60.8 ± 1.1
	Yearling	Merino	shoulder	58.2 ± 1	59.4 ± 0.9	63.2 ± 0.9	64.3 ± 0.9
			leg	57.4 ± 1	57.7 ± 0.9	61.4 ± 0.9	62.1 ± 0.9
Katanning	Lamb	Maternal	shoulder	64.5 ± 3.4	63.6 ± 3.1	67.5 ± 2.9	67.8 ± 3
			leg	60.6 ± 3.4	58.5 ± 3.1	64.0 ± 2.9	64.4 ± 3
		Merino	shoulder	66.3 ± 2.1	66.1 ± 1.9	70.7 ± 1.8	72.0 ± 1.9
			leg	62.9 ± 2.1	62.6 ± 1.9	65.4 ± 1.8	66.3 ± 1.9
		Terminal	shoulder	65.0 ± 1.3	64.6 ± 1.2	69.4 ± 1.1	70.6 ± 1.1
			leg	63.6 ± 1.3	63 ± 1.2	66.9 ± 1.1	67.1 ± 1.1
	Yearling	Merino	shoulder	59.0 ± 1.3	60.4 ± 1.2	64.7 ± 1.1	65.4 ± 1.1
			leg	57.9 ± 1.3	59.3 ± 1.2	62.7 ± 1.1	62.2 ± 1.1

**Table 5:** Partial correlation coefficients for tenderness, juiciness, liking of flavour and overall liking for the shoulder (above diagonal) and leg (below diagonal) samples for Chinese consumers.

Sensory Traits	Tenderness	Juiciness	Flavour	Overall Liking
Tenderness		0.81	0.73	0.77
Juiciness	0.79		0.74	0.77
Flavour	0.71	0.72		0.92
Overall liking	0.76	0.76	0.92	

## Data Availability

All related data and methods are presented in this paper. Additional inquiries should be addressed to the corresponding author.
